# Cardiac valve calcification and risk of cardiovascular or all-cause mortality in dialysis patients: a meta-analysis

**DOI:** 10.1186/s12872-018-0747-y

**Published:** 2018-01-25

**Authors:** Zhe Wang, Aili Jiang, Fang Wei, Haiyan Chen

**Affiliations:** Department of Blood Purification, The Second Hospital of Tianjin Medical University, Hexi District, 23 Pingjiang Road, Tianjin, 300211 China

**Keywords:** Cardiac valve calcification, Dialysis, Chronic kidney disease, Mortality, Meta-analysis

## Abstract

**Background:**

Vascular calcification is a risk factor for the pathogenesis of cardiovascular disease and mortality in dialysis patients. Nevertheless, the association between cardiac valve calcification (CVC) and the outcome of dialysis is still illusive. The purpose of this meta-analysis is to evaluate the association between theCVC and cardiovascular or all-cause mortality in dialysis patients.

**Methods:**

Literatures involving the baseline CVC and cardiovascular or all-cause mortality in dialysis patients were searchedfrom the PubMed, Embase, as well as two Chinese databases (i.e. Wanfang and CNKI databases). Articles published before November 2016were eligible to the study.

**Results:**

Ten studies involving 2686 participants were included. CVC was correlated with increased risk of cardiovascular mortality (hazard risk [HR]: 2.81; 95% confidence intervals [CI]: 1.92–4.10) and all-cause mortality (HR: 1.73; 95% CI: 1.42–2.11). Subgroup analysis showed an excess risk of all-cause mortality (HR: 1.35; 95% CI: 1.02–1.79) among patients with one CVC, and increased risk of all-cause mortality in patients with two CVCs (HR 2.15; 95% CI 1.57–2.94).

**Conclusions:**

CVC is correlated with higher cardiovascular and all-cause mortality risk in dialysis patients. Regular follow-up monitoring of CVC may be helpful for risk stratification of patients underwent dialysis.

## Background

Cardiovascular calcification, aprevalent condition at all stages of chronic kidney disease (CKD), is one of the major causes for increased cardiovascularmorbidity and mortality world wide [1]. It has been well acknowledged that cardiovascular calcification is associated with the pathogenesis of severe coronary artery disease, arterial stiffness, and peripheral vascular disease. The prevalence of vascular calcification usually increasedin patients with advanced CKD. For example, the prevalence in patients with stage 5 CKD on dialysis (stage 5D CKD) was superior to that of patients with stage 3D CKD (80–90% vs. 40%) [2].

Vascular calcification has been acknowledged as a strong predictor for cardiovascular morbidity and mortality inCKD population [3]. A cross-sectional analysis showed that cardiac valve calcification (CVC) was closely associated with vascular calcification that was considered as a risk factor for prevalent hemodialysis [4]. According to the previous cohort studies [5, 6], the association between CVC and mortality in dialysis patients is still controversial. Besides, thesample size of the existing cohort CVC studies on dialysis is small, which results in various differences among the studies. This meta-analysis was performed to investigate the association between CVC and risk of cardiovascular or all-cause mortality in dialysis patients.

## Methods

### Data sources

Meta-analysis was performed according to the recommendations proposed by the Preferred Reporting Items for Systematic Reviews and Meta-analyses Statement [7]. We searched the PubMed, Embase, as well as two Chinese databases including Wanfang and China National Knowledge Infrastructure (CNKI) databases using the following terms: ‘hemodialysis’ OR ‘peritoneal dialysis’ OR ‘end stage renal disease’ AND ‘cardiac valve calcification’ OR ‘valve calcification’ AND ‘mortality’ OR ‘death’. Studies published up to November 25, 2016 were eligible. Meanwhile, the references of selected articles were searched manuallyfor additional eligible studies.

### Inclusion criteria

Studies met the following criteria were eligible: 1) original observational cohort studies; 2) involving participants with end-stage kidney disease underwent dialysis; 3) evaluating the association between the presence and extent of CVC at baseline and subsequent cardiovascular or all-cause mortality; and 4) reporting outcomesas raw data or risk estimation of cardiovascular or all-cause mortality. In cases of several publications from the same study group, only the most recent complete publication was selected. Studies of cross-sectional design, published in types of review or protocol, or duplicated publication were excluded from the study.

### Data extraction

Two authors (Z Wang and HY Chen) independently collected data from the eligible studies, and did the data extraction including first author’s name, publication year, research design, geographical region of study, demographic characteristics of patients, examination methods, prevalence of CVC, number of death events, adjusted hazard ratio (HR) and 95% confidence intervals (CI), length of follow-up, and adjustment for covariates. A profound discussion was held in cases of any discrepancies during the data collection until consensus. The Newcastle–Ottawa Scale (NOS) for cohort studies was applied to assess the quality of methodology of each study [8]. A study with six or more stars was regarded as a higher quality study after NOS evaluation.

### Statistical analyses

We pooled the overall risk estimates using the adjusted HR with the 95% CI. The Cochran’s Q test and I^2^ statistic were utilized to evaluate the heterogeneity among studies. Random effect model as described by DerSimonian and Laird was applied in cases of significant heterogeneity (*P* < 0.10 and I^2^ > 50%). Otherwise, a fixed-effect model as described by Mentel-Haenszel was applied [9]. Subgroup analyses were performed by study region (Asia or non-Asian regions), dialysis modality (hemodialysisor peritoneal dialysis), and number of CVC (one or two calcified cardiac valve). The source of heterogeneity was analyzed by Meta-regression analysis. The publication bias was evaluated using the Begg’s and Egger’s [10] tests. *P* < 0.05 was considered to be statistically significant. The sensitivity analysis was carried out by excluding one study at each turn to test the robustness of the pooled results. Stata 11.0 software was used for the statistical analysis.

## Results

### Study selection

**Fig. 1:**
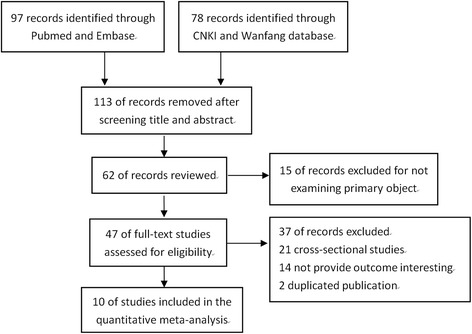
Flow chart of article selection

In total, 175 studies were initially identified through electronic search, among which 113were excluded as these articles were presented in the forms of meeting abstract, reviews or articles with no relevant outcomes reported. Then 52 studies were excluded mainly due to not confirming with the inclusion criteria. Finally, ten cohort studies involving 2686 dialysis patients [5, 6, 11–18] were eligible in this study. Flow chart of study selection was detailed in Fig. 1.

The baseline characteristics of the studies were listed in Table 1. All the ten studies were of a prospective design. The length of follow-up was 1.46-10 years. Echocardiographywas used to detect CVC.

**Table 1:** Study characteristics

Study	Region	Design	Patients (%male)	Age (years)	Detection methods	Prevalence of CVC	Comparison of CVC	Events number HR (95% CI)	Follow-up (years)	Ajustment for covariates	NOS
Takahashi 2013 [5]	Japan	Prospective study	HD 1290 (64.3)	61 ± 13	Echocardiography	57.50%	No. of CVC vs. absence	All-cause death (335): 1.47 (1.05-2.08), one CVC:1.43 (1.02-2.00), two CVC: 2.16 (1.51-3.11); Cardiovascular death (156):2.09 (1.17-3.94), one CVC1.68 (1.01-2.83), two CVC:2.80 (1.63-4.81)	10	Age, diabetes, BMI, albumin, creatinine, CRP and LVEF	8
Raggi 2011 [11]	USA	Prospective study	HD 144 (49.3)	55.4 ± 14.6	Echocardiography, EBCT	57.60%	No. of CVC vs. absence	All-cause death (59); one CVC:1.06 (0.54- 2.08), two CVC:2.12 (1.12 - 4.01)	5.6	Age, race, gender, diabetes mellitus status, history of atherosclerotic coronary vascular disease and pulse pressure	7
Wang 2003 [12]	China	Prospective study	PD 192 (51)	60 ± 10 (CVC); 53 ± 13 (no CVC)	Echocardiography	32.30%	Presence vs. absence	All-cause death (46):2.50 (1.32 to 4.76); Cardiovascular death (23):5.39 (2.16 to 3.48)	1.49	Age, male gender, dialysis vintage, diabetes and atherosclerotic	7
Panuccio 2004 [6]	Italy	Prospective study	HD 202 (55.9)	65.0 ± 10.6 (CVC); 57.1 ± 15.5 (no CVC)	Echocardiography	23.27%	Presence vs. absence	All-cause death (96):1.20 (0.75-1.92); Cardiovascular death (66):1.48 (0.86-2.54)	3.67	Age, sex, diabetes, CRP, ADMA, and background CV complications	7
Varma 2005 [13]	USA	Prospective study	HD 137 (54.7)	63 ± 15	Echocardiography	47.40%	Presence vs. absence	All-cause death (59):2.48 (1.49-4.13)	3.5	Not provided	5
Mohamed 2013 [14]	USA	Prospective study	101 (67.3)	57.7 ± 9.2 (CVC); 46.7 ± 12.9 (no CVC)	Echocardiography, MSCT	35.64%	One CVC vs. absence	All-cause death (11): 1.37 (0.62-3.05)	2.85	Age, gender, and IL-6	7
Li 2016 [15]	China	Prospective study	HD 302 (53.6)	60.9 ± 12.9 (CVC); 55.9 ± 15.8 (no CVC)	Echocardiography	32.78%	Presence vs. absence	All-cause death (63):1.88 (1.11-3.19); Cardiovascular death (36): 3.47 (1.76-6.84)	2	Age, diabetes, beta- blocker, ACEI or ARB, pre-HD DBP, serum phosphorus, serum albumin, CRP, uric acid, LV systolic dysfunction, and history of CV events and HVC.	8
Zhong 2011 [16]	China	Prospective study	HD 96 (57.3)	61 ± 14 (CVC); 52 ± 8 (no CVC)	Echocardiography	32.29%	Presence vs. absence	Cardiovascular death(12): 3.50 (2.23~ 5.52)	1.46	Age, gender, duration of dialysis, diabetes, atherosclerotic vascular disease, and CRP	6
Wang 2014 [17]	China	Prospective study	PD 112 (61.6)	71.57 ± 9.52 (CVC); 56.15 ± 15.28 (no CVC)	Echocardiography	Not provided	Presence vs. absence	All-cause death (26): 3.139 (1.181-8.345)	4.18	Age, diabetes, calcium, phosphorus, rGFR, CRP, and PA	8
Chen 2016 [18]	China	Prospective study	HD 110 (58.2)	55.2 ± 1.4	Echocardiography	25.50%	Presence vs. absence	All-cause death (25): 1.563 (0.637–3.836); Cardiovascular death (16): 3.80 (1.15-12.558)	3.5	Age, gender, albumin, AAC, and 25(OH)D	7

*CVC* cardiac valve calcification, *HR* hazard ratio, *95% CI* 95% confidence intervals, *NOS* Newcastle–Ottawa Scale, *HD* hemodialysis, *PD* peritoneal dialysis, *BMI* body mass index, *CRP* C-reactive protein, *LVEF* left ventricular ejection fraction, *CV* cardiovascular, *ADMA* asymmetric dimethyl arginine, *IL-6* interleukin-6, *ACEI* angiotensin converting enzyme inhibitors, *ARB* angiotensin receptor blocker, *DBP* diastolic blood pressure, *LV* left ventricular, *rGFR* residual glomerular filtration rate, *PA* prealbumin, *AAC* aortic arch calcification, *EBCT* electron beam computerized tomography, *MSCT* multislice computed tomography

### All-cause and cardiovascular mortality

**Fig. 2:**
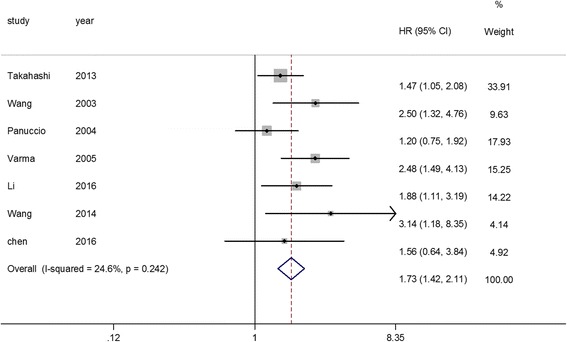
Association between CVC and all-cause mortality risk revealed by Forest plot

**Fig. 3:**
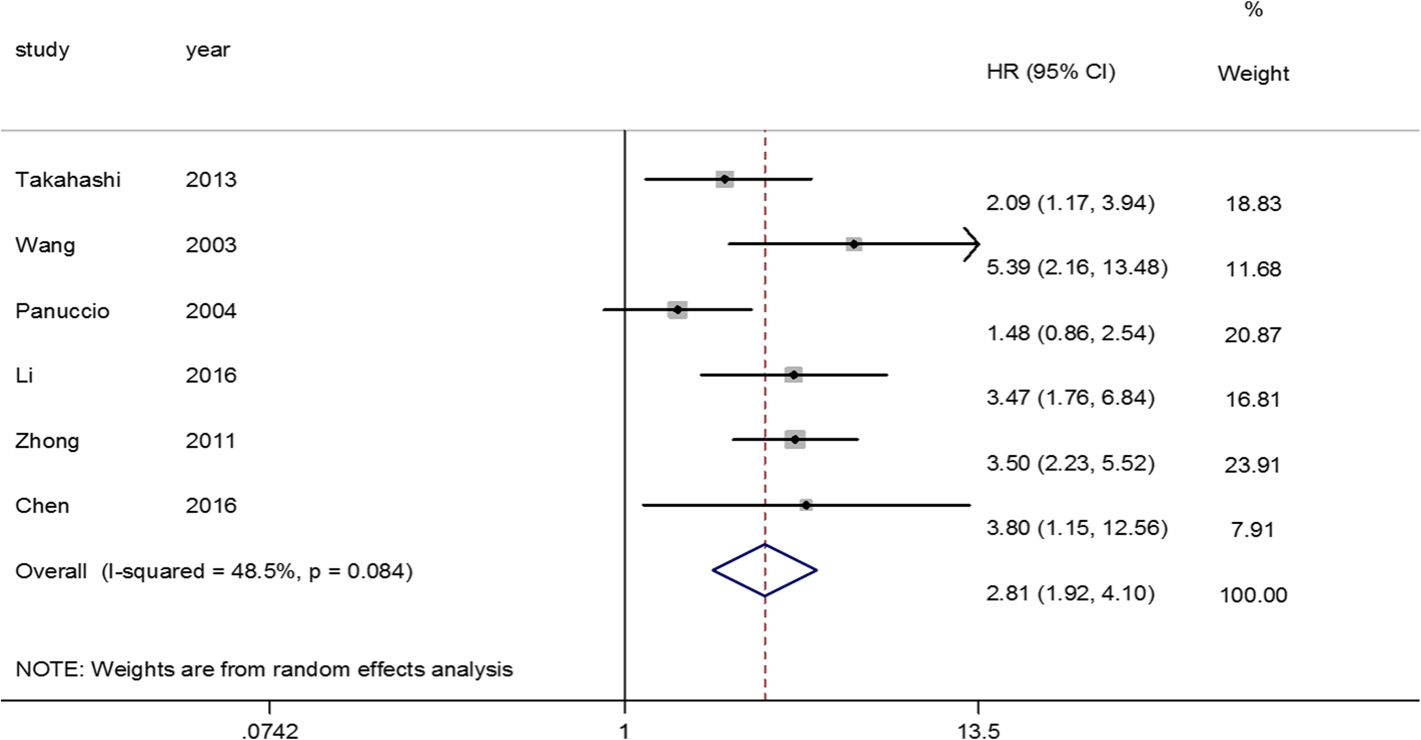
Association between CVC and cardiovascular mortality risk revealed by Forest plot

A total of 650 all-cause mortality events were reported in seven studies [5, 6, 12, 13, 15, 17, 18] among the 2345 dialysis patients. Three studies [5, 11, 14] reported the all-cause mortality risk among patients with one calcified cardiac valve compared with those without CVC. Two studies [5, 11] reported the all-cause mortality risk among patients with two calcified cardiac valve compared with those without CVC (Table 2). CVC was related to a greater risk of all-cause mortality (HR: 1.73; 95% CI: 1.42–2.11; I^2^ = 24.6%; *P* = 0.242, Fig. 2) in a fixed-effect model. A total of 309 cardiovascular mortality events were reported in six studies [5, 6, 12, 15, 16, 18] among the 2192 dialysis patients. CVC was associated with 1.81-fold greater risk of cardiovascular mortality (HR: 2.81; 95% CI: 1.92–4.10; I^2^ = 48.5%; *P* = 0.084, Fig. 3) in a random effect model.

**Table 2:** Subgroup analyses of all-cause and cardiovascular mortality

Subgroups	Number of studies	Number of patients	Pooled HR	95% CI	Heterogeneity among studies
All-causemortality					
Region					
Asia	5	2006	1.76	1.38–2.25	*P* = 0.450;I^2^ = 0.0%
Non-Asian region	2	339	1.71	0.84–3.49	*P =* 0.040;I^2^ = 76.3%
Dialysis modality					
Hemodialysis	5	2041	1.62	1.30–2.00	*P* = 0.301;I^2^ = 17.8%
Peritoneal dialysis	2	304	2.68	1.57–4.58	*P* = 0.703;I^2^ = 0.0%
Follow-up duration					
≥ 2 years	6	2153	1.67	1.35–2.05	*P* = 0.255;I^2^ = 23.8%
< 2 years	1	192	2.50	1.32–4.76	–
Echocardiography					
1 physician	4	743	2.320	1.714–3.140	*P* = 0.778;I^2^ = 0.0%
2 physicians	3	1602	1.386	1.064–1.805	*P* = 0.762;I^2^ = 0.0%
Number of CVC					
1	3	1535	1.35	1.02–1.79	*P* = 0.738;I^2^ = 0.0%
2	2	1434	2.15	1.57–2.94	*P* = 0.960;I^2^ = 0.0%
Cardiovascular mortality					
Region					
Asia	5	1990	3.26	2.43–4.36	*P* = 0.492;I^2^ = 0.0%
Non-Asian region	1	202	1.48	0.86–2.54	–
Dialysis modality					
Hemodialysis	5	2000	2.57	1.96–3.36	*P* = 0.117;I^2^ = 45.8%
Peritoneal dialysis	1	192	5.39	2.16–3.48	–
Follow-up duration					
≥ 2 years	4	1904	2.17	1.56–3.03	*P* = 0.202;I^2^ = 35.0%
< 2 years	2	288	3.81	2.54–5.72	*P* = 0.407;I^2^ = 0.0%
Echocardiography					
1 physician	3	590	3.718	2.624–5.268	*P* = 0.691;I^2^ = 0.0%
2 physicians	3	1602	1.890	1.256–2.845	*P* = 0.333;I^2^ = 9.0%
Number of CVC					
1	1	1290	1.68	1.01–2.83	–
2	1	1290	2.80	1.63–4.81	–

*CVC* cardiac valve calcification

### Subgroup analyses, meta-regression and sensitivity analyses

Subgroup analysis for the study region demonstrated thatAsian patients with CVC had greater cardiovascular (HR: 3.255; 95%CI: 2.428-4.363; I^2^ = 0.0%, *P* = 0.492) and all-cause mortality (HR: 1.761; 95%CI: 1.380-2.246; I^2^ = 0.0%,*P* = 0.45). However, in the non-Asian region, no relationship was identified between presence of CVC and cardiovascular or all-cause mortality. Subgroup analyses based on the number of CVC, dialysis modality, and length of follow-upshowed the cardiovascular or all-cause mortality was higher than those without CVC. Subgroup analysis was performed with the number of physiciansanalyzing echocardiographic recordings serving as a variable, which revealed significant decrease in the heterogeneity (all-cause mortality: two physicians: HR: 1.386; 95% CI: 1.064–1.805; I^2^ = 0.0%; *P* = 0.762; one physician: HR: 2.320; 95% CI: 1.714–3.140; I^2^ = 0.0%; *P* = 0.778; cardiovascular mortality: two physicians: HR: 1.890; 95% CI: 1.256–2.845; I^2^ = 9.0%; *P* = 0.333; one physician: HR: 3.718; 95% CI: 2.624–5.268; I^2^ = 0.0%; *P* = 0.691, Table 2). In the Meta-regression analysis, region, follow up duration, dialysis modality, being a multicenter study or not, a randomized study or not, a blinded follow up or not served as variables to investigate the effects of CVC on the cardiovascular or all-cause mortality. No statistical differences were noticed (all *P* > 0.1). In addition, Meta-regression analysis showed that there was atrend towards the number of physiciansanalyzing echocardiographic recordings being correlated tostudy outcomes (all-cause mortality: *P* = 0.054; cardiovascular or all-cause mortality: *P* = 0.061). Sensitivity analyses by excluding one study at each turn showed that there were no changes in the direction of pooling risk estimate of all-cause mortality (pooled HR: 1.62-1.88) and cardiovascular mortality (pooled HR: 2.41-3.25). For the all-cause mortality analysis, only one study (Wang et al., 2003) including 192 cases corrected the dialysis vintage (HR: 2.5, 95%CI: 1.32-4.76). The other five subsequent studies including 2153 subjects (HR: 1.665, 95%CI: 1.351-2.053; I^2^ = 23.8%; *P* = 0.255). For the cardiovascular mortality analysis, Wang et al. (2003) and Zhong and Na (2011) corrected the dialysis vintage. After a pooled analysis, the following data were obtained: HR: 3.810; 95% CI: 2.539–5.720; I^2^ = 0.0%; *P* = 0.407. The other four subsequent studies involving 1904 subjects were included in the analysis (HR: 2.265; 95% CI: 1.473–3.481; I^2^ = 35.0%; *P* = 0.202).

### Publication bias

**Fig. 4:**
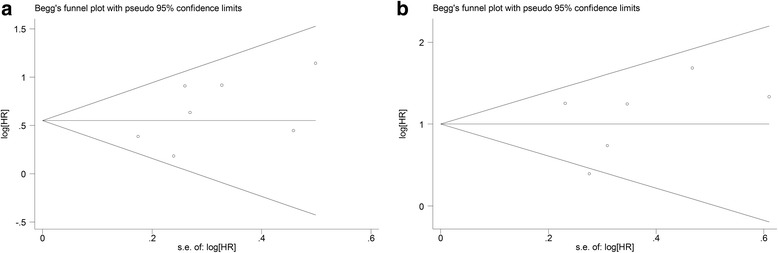
Evaluation of publication bias for all-cause mortality (**a**) andcardiovascular mortality (**b**)

Begg’s rank correlation test and Egger’s linear regression test showed nopublication bias for all-cause mortality (Begg’s test: *P* = 0.368, Fig.4a; Egger’s test: *P* = 0.199). Besides, no evidence was observed in the publication bias of the cardiovascular mortalityaccording to the Begg’s rank correlation test (*P* = 0.707, Fig.4b) and Egger’s linear regression test (*P* = 0.517).

## Discussion

This meta-analysis showed that prevalence of aortic arch calcification ranged from 23.27% to 57.60%, and presence of CVC increased the risk of cardiovascular mortality by 181% and all-cause mortality by 73% in dialysis patients. Kidney Disease Improving Global Outcome (KDIGO) indicates that detection of vascular/valve calcification is useful for risk stratification of patients undergoing dialysis, while echocardiographyis reasonable for the detection of valve calcification [19]. Our study further supports the recommendations of KDIGO.

Subgroup analysis revealed CVC in dialysis patients with two calcified cardiac valves was associated with greater mortality. The results supported that higher degree of CVC was associated with increased risk of mortality. Additionally, peritoneal dialysis patients with presence of CVC seemed to show a higher risk of mortalitythan hemodialysis patients. CVC was related to higher mortality riskin Asian patients. Moreover, single physiciananalyzing echocardiographic recordingswas related to higher mortality risk.

The inter-study heterogeneity showed decrease after grouping based on the hemodialysis (I^2^ = 17.8%) and peritoneal dialysis (I^2^ = 0.0%) in the all-cause mortality. The cardiovascular and all-cause mortality in the peritoneal dialysis patient with concurrent cardiac valve calcification was higher than that of the hemodialysis patients. Besides the heterogeneity, further studies are needed to investigate the effects of dialysis mode on the effects of vascular calcification and prognosis. In the all-cause mortality analysis, the risk of cardiac valve calcification induced all-cause mortality in the Asian population was higher than that of the other populations, among which in a previous study by Panuccio et al. (2004) in the non-Asian population [HR (95% CI):1.20 (0.75-1.92)] that was significantly lower than the other studies. This may be related to the exclusion of heart failure subjects, which may be an important cause for the mortality. For the sensitivity analysis, the heterogeneity showed significant decrease after excluding the study reported by Varma et al. (I^2^ = 12.4%), which may be related the higher heterogeneity as the study was of low quality. Meta-regression analysis revealed region, follow up duration, dialysis modality, being a multicenter study or not, a randomized study or not, a blinded follow up or not were not the major sources for the inter-study heterogeneity (all *P* > 0.1). Besides, the number of physiciansanalyzing echocardiographic recordings served as variable, which also showed no statistical differences (*P* = 0.054). As only a few studies were included, it may be a source for the heterogeneity despite a *P* value of > 0.05. For further analysis, subgroup analysis was performed using the number of physicians as the variable, which revealed significant decrease in the heterogeneity. Thus, single physician may increase the bias, which overestimated the effects of CVC on the all-cause mortality.

For the analysis of cardiovascular mortality, the regions were divided into Asia and non-Asian regions. The heterogeneity in the 5 studies performed in Asia was low (I^2^ = 0.0%). Only one study (Panuccioet al, 2004) was performed in the non-Asian region, which may be responsible for the heterogeneity. This may be related to the exclusion of patients with heart failure that was an important cause for cardiovascular mortality. Meta-regression analysis revealed region, follow up duration, dialysis modality, being a multicenter study or not, a randomized study or not, a blinded follow up or not were not the major sources for the inter-study heterogeneity (all *P* > 0.1). Besides, the number of physiciansanalyzing echocardiographic recordings served as variable (*P* = 0.061). As only a few studies were included, it may be a source for the heterogeneity despite a *P* value of > 0.05. For further analysis, subgroup analysis was performed using the number of researchers as the variable, which revealed significant decrease in the heterogeneity (two physicians: HR: 1.890; 95% CI: 1.256–2.845; I^2^ = 9.0%; *P* = 0.333; one physician: HR: 3.718; 95% CI: 2.624–5.268; I^2^ = 0.0%; *P* = 0.691). Thus, single physician may increase the bias, which overestimated the effects ofCVC on the cardiovascular mortality. In a previous study, Panuccio et al. (2004) excluded the patients with heart failure, which may increase the heterogeneity. Meta-regression analysis showed heart failure may be a source for the heterogeneity (*P* = 0.064). In the meta-analysis excluding such study, the heterogeneity showed significant decrease (HR: 3.255; 95% CI: 2.428–4.363; I^2^ = 0.0%; *P* = 0.492). As heart failure was one of the major causes for the cardiovascular mortality, exclusion of heart failure may underestimate the effects of CVC.

CVC isan active pathobiological process resulting in calcium deposition involving aging, inflammation, CKD, diabetes mellitus, and phenotypic switch of vascular smooth muscle cells. In an environment with changed profiles of calcification-regulating humoral factors including calcium and phosphate, VSMCs can transform to osteo/chondrogenic phenotype. In addition, other factors such as degradation of elastin fibers also played important roles insubsequent calcification [20].

CKD progression may trigger various abnormalities in mineral and bone metabolism such as hyperphosphatemia, hypercalcemia, secondary hyperparathyroidism, bone disorders, and cardiovascular calcification. To date, several factors were identified as the risk factors of cardiovascular calcification, including metabolic disorder of calcium, phosphorus, parathyroid hormone, and fibroblast growth factor 23, inflammation, oxidative stress, declining of calcification inhibitor such as fetuin-A and matrix Gla protein, as well as pharmacological interventions such as active vitamin D and calcium-based phosphate binders. These factors may increase the cardiovascular events and mortality finally.

CVC refers to a condition with the presence of bright echoes of more than 1 mm on one or more cusps of the aortic valve, mitral annulus or mitral valve based on the guidelines of the American Society of Echocardiography [21]. Generally, dialysis patients usually present CVC [22]. The incidence and progression of CVC increased with the duration of dialysis [23]. In a previous study, CVC patients showed elevation of left ventricular mass index and pulmonary artery pressure and decrease of ejection fraction [24]. Besides, CVC was associated with the arterial wall stiffness, which then resulted in cardiac afterload elevation and left ventricular hypertrophy that were considered to be markers for the decrease of heart function [25]. Furthermore, CVC was significantly associated with peripheral arterial calcification, alterations of mineral metabolism [26], coronary artery calcification [27], arterial calcification [28], carotid atherosclerosis, arrhythmias, stroke and mortality [11]. Therefore, it was necessary to evaluate the CVC of the patients, which contributed to the risk stratification, modulation of treatment regimen, as well as delaying the calcification. In a previous meta-analysis [29], patients assigned to non-calcium-based binders had a 22% reduction in all-cause mortality compared with those assigned to calcium-based phosphate binders, and the reduction in vascular calcification was greater in patients assigned to non-calcium-based phosphate binders than in those assigned to calcium binders. The KDIGO 2017 clinical practice guideline update for CKD-mineral and bone disorder (CKD-MBD) [30] suggests restricting the dose of calcium-based phosphate binders. We need to use calcium-based phosphate binders or non-calcium-based phosphate binders, calcitriol or vitamin D analogs, and calcimimetics in a reasonable manner, in order to avoid the increased calcium load and the poor calcium phosphate control, prevent or retard the progression of cardiovascular calcification in dialysis patients.

Our study had some limitations. Firstly, some eligible studies were of small sample size and short follow-up duration, which may lead to limited generalizability. Secondly, the adjust covariates in different studies were not the same, which might affect the results of this meta-analysis. Thirdly, there are some negative findings that are not published, which may affect the results of this meta-analysis. Finally, the result based on the limited studies may be not robust, especially in the subgroup analyses.

## Conclusion

In summary, this meta-analysis indicates that CVC is associated with higher all-cause and cardiovascular mortality in dialysis patients. The number of calcified cardiac valve is correlated with the mortality risk. Our study provides the evidence that detection of valve calcification is beneficial for risk stratification of dialysis. In future, more prospective trials with large sample size are needed to further evaluate the relationship between CVC and the outcome of dialysis patients.

## Abbreviations

CI: Confidence intervals
CKD: Chronic kidney disease
CNKI: China national knowledge infrastructure
CVC: Cardiac valve calcification
HR: Hazard risk
NOS: Newcastle–Ottawa Scale

## References

[1] Bover J, Ureña-Torres P, Górriz JL (2016). Cardiovascular calcifications in chronic kidney disease: potential therapeutic implications. Nefrologia.

[2] Karohl C, D'MarcoGascón L, Raggi P (2011). Noninvasive imaging for assessment of calcification in chronic kidney disease[J]. Nat Rev Nephrol.

[3] Yamada S, Giachelli CM. Vascular calcification in CKD-MBD: roles for phosphate, FGF23, and Klotho. Bone. 2016 Nov;12 [Epub ahead of print] 10.1016/j.bone.2016.11.012 PMC5429216 27847254

[4] Bellasi A, Ferramosca E, Ratti C (2012). Cardiac valve calcification is a marker of vascular disease in prevalent hemodialysis patients. J Nephrol.

[5] Takahashi H, Ishii H, Aoyama T (2013). Association of cardiac valvular calcifications and C-reactive protein with cardiovascular mortality in incident hemodialysis patients: a Japanese cohort study. Am J Kidney Dis.

[6] Panuccio V, Tripepi R, Tripepi G (2004). Heart valve calcifications, survival, and cardiovascular risk in hemodialysis patients. Am J Kidney Dis.

[7] Moher D, Liberati A, Tetzlaff J (2009). Preferred reporting items for systematic reviews and meta-analyses: the PRISMA statement. J ClinEpidemiol.

[8] Liu G, Long M, Hu X (2015). Effectiveness and safety of warfarin in dialysis patients with atrial fibrillation: a meta-analysis of observational studies. Medicine (Baltimore).

[9] Higgins JP, Thompson SG (2002). Quantifying heterogeneity in a meta-analysis. Stat Med.

[10] Egger M, Smith GD, Altman DG (2001). Systematic reviews in health care: meta-analysis in context.

[11] Raggi P, Bellasi A, Gamboa C (2011). All-cause mortality in hemodialysis patients with heart valve calcification. Clin J Am SocNephrol.

[12] Wang AY, Wang M, Woo J (2003). Cardiac valve calcification as an important predictor for all-cause mortality and cardiovascular mortality in long-term peritoneal dialysis patients: a prospective study. J Am SocNephrol.

[13] Varma R, Aronow WS, McClung JA (2005). Prevalence of valve calcium and association of valve calcium with coronary artery disease, atherosclerotic vascular disease, and all-cause mortality in 137 patients undergoing hemodialysis for chronic renal failure. Am J Cardiol.

[14] Mohamed BA, Yang W, Litt H (2013). Valvular calcification, inflammation, and mortality in dialysis patients. J Heart Valve Dis.

[15] ZL LI, He CS, Yh C (2016). Association of heart valve calcification with cardiovascular outcomes in patients on maintenance hemodialysis. J South Med Univ.

[16] Zhong B, Na Y (2011). Influence of cardiac valve calcification to all-cause mortality in chronic hemodialysis patients. J Nephrol Dialy Transplant.

[17] Wang CY. Risk factor analysis of calcification in aortic and mitral valves and survival in maintenance peritoneal dialysis patients[D].Su Zhou: Su Zhou University. 2014:1-48. 10.1159/000355729 24247761

[18] Chen XN, Chen ZJ, Ma XB (2015). Aortic artery and cardiac valve calcification are associated with mortality in Chinese hemodialysis patients: a 3.5 years follow-up. Chin Med J.

[19] KDIGO (2009). Clinical practice guideline for the diagnosis, evaluation, prevention, and treatment of chronic kidney disease-mineral and bone disorder (CKD-MBD). Kidney Int Supp.

[20] Vervloet M, Cozzolino M (2017). Vascular calcification in chronic kidney disease: different bricks in the wall?. Kidney Int.

[21] Choi MJ, Kim JK, Kim SG (2013). Association between cardiac valvular calcification and myocardial ischemia in asymptomatic high-risk patients with end-stage renal disease. Atherosclerosis.

[22] Ribeiro S, Ramos A, Brandao A (1998). Cardiac valve calcification in haemodialysis patients: role of calcium-phosphate metabolism. Nephrol Dial Transplant.

[23] Gallieni M, Caputo F, Filippini A (2012). Prevalence and progression of cardiovascular calcifications in peritoneal dialysis patients: a prospective study. Bone.

[24] Sayarlioglu H, Acar G, Sahin M (2013). Prevalence and risk factors of valvular calcification in hemodialysis patients. Iran J Kidney Dis.

[25] Strózecki P, Odrowaz-Sypniewska G, Manitius J (2005). Cardiac valve calcifications and left entricular hypertrophy in hemodialysis patients. Ren Fail.

[26] Ribeiro S, Ramos A, Brandao A (1998). Cardiac valve calcification in haemodialysis patients: role of calcium- phosphate metabolism. Nephrol Dial Transplant.

[27] Kitamura K, Fujii H, Nakai K, et al. Relationship between cardiac calcification and left ventricular hypertrophy in patients with chronic kidney disease at hemodialysis initiation. Heart Vessel. 2017;21 [Epub ahead of print] 10.1007/s00380-017-0969-4 28324126

[28] Bellasi A, Ferramosca E, Ratti C (2012). Cardiac valve calcification is a marker of vascular disease in prevalent hemodialysis patients. J Nephrol.

[29] Jamal SA, Vandermeer B, Raggi P (2013). Effect of calcium-based versus non-calcium-based phosphate binders on mortality in patients with chronic kidney disease: an updated systematic review and meta-analysis. Lancet.

[30] Ketteler M, Block GA, Evenepoel P (2017). Executive summary of the 2017 KDIGO chronic kidney disease-mineral and bone disorder (CKD-MBD) guideline update: what's changed and why it matters. Kidney Int.

